# On the Protective Power of Vaccination

**Published:** 1852-01

**Authors:** S. Annan


### ARTICLE V.

*On the Protective Power of Vaccination.* By S. Annan, M. D.

The following view upon this subject, have recently been promulgated, at a meeting ot the Royal Medical and Chirurgical Society of London.

Dr. Gregory, the Physician of the Small-pox Hospital, stated, that from the year 1844 to 1850, 2854 cases of small-pox had been admitted ; and that of these, 1500 were after vaccination. The whole number of deaths had been 579; and of these only 75 were amongst the vaccinated portion. He also gave his opinion that the protection afforded by vaccination up to the period of puberty, fifteen years of age, was equal to that afforded by inoculation for small-pox all through life. After fifteen, the system was subjected to another law. Previous to that age, we might banish all fear ; inasmuch as cases of small-pox after vaccination were exceedingly rare ; but after that period, vaccinated persons were liable to a first attack of the disease, and were exposed to the chances of a second attack at fifty or sixty years of age. Inoculation gave one attack of small-pox, and there was an end of it. With a few rare exceptions, in the cases of particular families and individuals, there was no second and third attack. Modified small-pox was unknown up to the year 1817, about fifteen years after vaccination was first performed. The cases of this disease had gone on increasing since the year 1825, and the results now were, that 1500 patients had been admitted into the London Small Pox Hospital, in seven years, with small-pox after vaccination. Most of these were modified, but many were of a severe form of the disease. The deaths from small-pox after vaccination, might be said to be a little above five per cent; in some places, as at Copenhagen, it was as low as three per cent. He had never seen modified small-pox in the young, either in public or private practice ; neither had he read of it in books, nor heard of its occurrence abroad. It was only to be found occurring in the adult. He hoped the statements advanced would not shake the confidence of the public in vaccination. Even if vaccination prevented small-pox only in one-half of the cases in which it was performed, it was a great protection ; and as it was shown by statistics that about half the population died before the age of fifteen, it afforded to that half, at least, perfect and complete protection.

Drs. Mayo and Copeland were of opinion, as the result of their experience, that the protective power of vaccination, until the age of fourteen, was more complete than that of inocculation ; that a greater number became affected with small-pox after inoculation, than after vaccination ; and that more of the inoculated died than of the vaccinated.

Dr. Gregory also stated, that he regarded re-vaccination as a proceeding of very little moment. It satisfied the mind of the public, but did not affect any real good. It was an error to suppose it afforded any additional protection. After fifteen years of age, the constitution began to be susceptible, for the first time, to the influence of small-pox, and the susceptibility increased up to middle age and muturity. There was but one mode of adding to the protective power of the first vaccination, and that was by inocculation with small-pox matter after the age of fifteen. Cazenave and others, had performed in France, many experiments, which proved, that inoculation after fifteen, in persons previously vaccinated, did not produce a vesicular or pustular eruption, but only a popular one, and that this was not contagious. This he knew to be true, and he firmly believed it served as a protection for life.

With respect to the indications afforded by the appearance of the cicatrix, as to the perfect or the imperfect performance of vaccination, Dr. Gregory remarked, that he thought it had been long ago conclusively settled, that no conclusion whatever could be drawn from the appearance of the cicatrix. If a good cicatrix were found, then you might be satisfied that the vaccination had been perfectly performed ; but if the cicatrix was imperfect, you had no right to assume that the patient had not been well vaccinated ; for in these cases, the process of reparation might have been quick, there might have been little inflammation, or there might be other causes to account for the imperfect cicatrix. He had long long ago published cases on this point.

Dr. Addison inquired of Dr. Gregory, what his impression was respecting the identity of small-pox and chicken pox?

Dr. Gregory answered that the diseases, though bearing some relation to each other, were undoubtedly different and distinct in their nature. In proof of this, it has been demonstrated, that genuine vaccination had been received before and the occurrence of chicken-pox. The occurrence of the latter previously, made no difference in the development of the former. In addition to this, the two diseases might go on together in the same person. None of these modifications had ever been witnessed in cases of small-pox and vaccination.

Dr. Marshall Hall stated, that sometimes a child resisted vaccination; and he desired to know from Dr. Gregory, whether it was still liable to take the small-pox? His own son had been vaccinated fourteen times without effect. No vaccine vesicle ever formed. At thirteen years of age, he was observed to be covered with an eruption. Some of the spots once exhibited the form of distended vesicles, of moderate size, observed in chicken-pox. Others of the spots went through the regular course of horn-pock, occupying five or six days. One or two on the face left distinct pits, the result of sloughing, as seen in small-pox. Dr. Hall added, that such a case seemed to demonstrate the insecurity of the patient, when vaccination had failed several times, and to confirm the opinion of Dr. Thompson, that varicella and modified small-pox were the same disease, for in it they occurred simultaneously. Dr. Gregory replied that he had no hesitation in saying that the variolous poison had done its worst; and though Dr. Hall's son might have the ill luck to contract secondary small-pox, the great probability was that he would not.

At the same meeting of the Society several cases were narrated, of second and third attacks of small-pox; some of them after inoculation, some after natural small-pox. In one of the cases a gentleman who had been vaccinated in infancy, when three months old, had three attacks of small-pox; one when he was six years old, a second when he was eleven, and he died in the East Indies; of the third attack, when he was twenty-three years of age.

This must be regarded as an extremely interesting discussion; partly from the nature of the subject, and partly because several of the most eminent physicians of Great Britain participated in it. It would appear that we are at last arriving at a proper knowledge and a just appreciation of the value of vaccination. While we must admit with Chomel, that "we cannot fairly exact more from vaccination than from the small-pox itself," if Dr. Gregory's opinions are well founded, that every individual after fifteen years of age, although fully protected up to that time by the power of vaccine disease, is liable to be attacked by small-pox, and that re-vaccination is no manner of service, there is great room for apprehension that as the general force of vaccination diminishes in large communities, the small-pox contagion may acquire an augmentation of power, and at length come to predominate. Dr. Gregory informs us, that the cases of small-pox after vaccination, have gone on increasing since 1825. If this increase should be progressive, as must necessarily happen under existing circumstances, it is manifest that in time, small-pox will preponderate, and the beneficial effects of vaccination wear out. As the force of general protection is lessened, the power of general predisposition will become greater, and we shall be compelled to resort to some additional means to prevent a return to the old epidemic ravages of small-pox.

At the present time, although we have occasional examples of persons dreadfully disfigured, and of death from small-pox after vaccination, the number is not so great as to cause much alarm. The improvement upon the old state of things is inestimable. In England, previous to the introduction of inoccu-lation, one-tenth of the total mortality was occasioned by small-pox. After inoculation was introduced, it fell to one-fourteenth; but now, when vaccination is general, the deaths by small-pox are about one in eighty-five from all diseases; thus amounting to only one-sixth of the ratio, when small-pox inoculution was the sole preventive.

If matters should threaten to become much worse, and the number of cases subsequent to vaccination, go on progressively increasing, we are not shut up to patient endurance of the evil, but can have recourse to inoculation after puberty, depending upon vaccination up to that perion. If the views at present entertained as to the mildness of small-pox communicated at that time, and as to its protective power being complete for the remainder of life, should be fully established by repeated trials, and by time, our situation will be far from being deplorable. In the mean while, each adult may hope that the protection in his own case, although not perfect, is sufficient to prevent an attack of this loathsome disease, or to modify it, if it does appear.—*Transylvania Med. Jour.*

